# Culturally-adapted Family Intervention (CaFI) for African-Caribbeans diagnosed with schizophrenia and their families: a feasibility study protocol of implementation and acceptability

**DOI:** 10.1186/s40814-016-0070-2

**Published:** 2016-08-03

**Authors:** Dawn Edge, Amy Degnan, Sarah Cotterill, Katherine Berry, Richard Drake, John Baker, Christine Barrowclough, Adwoa Hughes-Morley, Paul Grey, Dinesh Bhugra, Patrick Cahoon, Nicholas Tarrier, Shôn Lewis, Kathryn Abel

**Affiliations:** 1 Centre for Women’s Mental Health, Institute of Brain, Behaviour and Mental Health and School of Psychological Sciences, University of Manchester, G6 Coupland Building, Manchester, M13 9PL UK; 2 Centre for Women’s Mental Health, Institute of Brain, Behaviour and Mental Health and School of Psychological Sciences, University of Manchester, Third Floor Jean McFarlane Building, Manchester, UK; 3 Centre for Biostatistics, Institute of Population Health, University of Manchester, Manchester, UK; 4 School of Psychological Sciences, University of Manchester, Second Floor Zochonis Building, Manchester, UK; 5 Institute of Brain, Behaviour and Mental Health, University of Manchester, Third Floor Jean McFarlane Building, Manchester, UK; 6 Faculty of Medicine and Health, University of Leeds, Baines Wing, Leeds, LS2 9JT UK; 7 Institute of Psychiatry, Kings College London, London, WC2R 2LS UK; 8 Manchester Mental Health and Social Care NHS Trust, Chorlton House, Chorlton, Manchester, UK; 9 Centre for Women’s Mental Health, Institute of Brain, Behaviour and Mental Health, University of Manchester, Third Floor Jean McFarlane Building, Manchester, UK; 10 MRC North West Hub for Trials Methodology Research, Manchester Academic Health Science Centre, University of Manchester, Williamson Building, Manchester, UK; 11 York Trials Unit, Department of Health Sciences, University of York, York, UK; 12 Manchester Mental Health & Social Care Trust, 70 Manchester Rd, Manchester, M21 9UN UK

**Keywords:** Cultural adaptation, Family intervention, Psychological therapy, African-Caribbean, Black British, Black and minority ethnic (BME), Schizophrenia, Psychosis, Severe mental illness (SMI), Feasibility trial

## Abstract

**Background:**

African-Caribbeans in the UK have the highest schizophrenia incidence and greatest inequity in access to mental health services of all ethnic groups. The National Institute for Health and Care Excellence (NICE) highlights this crisis in care and urgent need to improve evidence-based mental healthcare, experiences of services and outcomes for this group. Family intervention (FI) is clinically and cost-effective for the management of schizophrenia but it is rarely offered. Evidence for FI with minority ethnic groups generally, and African-Caribbeans in particular, is lacking. This study aims to test the feasibility and acceptability of delivering Culturally-adapted Family Intervention (CaFI) to African-Caribbean service users diagnosed with schizophrenia.

**Methods/Design:**

This is a feasibility cohort design study. Over a 12-month intervention period, 30 service users and their families, recruited from hospital and community settings, will receive ten one-hourly sessions of CaFI. Where biological families are absent, access to the intervention will be optimised through ‘family support members’; trusted individuals nominated by service users or study volunteers.

We shall collect data on eligibility, uptake, retention and attrition and assess the utility and feasibility of collecting various outcome measures including readmission, service engagement, working alliance, clinical symptoms and functioning, perceived criticism, psychosis knowledge, familial stress and economic costs. Measures will be collected at baseline, post-intervention and at 3-month follow-up using validated questionnaires and standardised interviews. Admission rates and change in care management will be rated by independent case note examination. Variability in the measures will inform sample size estimates for a future trial. Independent raters will assess fidelity to the intervention in 10 % of sessions. Feedback at the end of each session along with thematically-analysed qualitative interviews will examine CaFI’s acceptability to service users, families and healthcare professionals.

**Discussion:**

This innovative response to inequalities in mental healthcare experienced by African-Caribbeans diagnosed with schizophrenia might improve engagement in services, access to evidence-based interventions and clinical outcomes. Successful implementation of CaFI in this group could pave the way for better engagement and provision across marginalised groups and therefore has potentially important implications for commissioning and service delivery in ethnically diverse populations. This study will demonstrate whether the approach is feasible and acceptable and can be implemented with fidelity in different settings.

## Background

### African-Caribbeans, schizophrenia and mental health services

Over several decades, increased incidence of schizophrenia and more negative experiences of mental healthcare have been consistently reported among people of African-Caribbean origin compared with other ethnic minorities in the UK [1–7]. Their care pathways are less likely to involve general practitioners (GPs) and frequently involve multiple help-seeking attempts [7]. This delays access to diagnosis and evidence-based treatment, increasing the duration of untreated symptoms and illness acuity on contact with services. African-Caribbeans’ admission to specialist mental health services often involves the criminal justice system and detention under the Mental Health Act 2007 [8, 9].

As inpatients, African-Caribbean people continue to experience more coercive care than other ethnic groups, including increased rates of seclusion, control and restraint, and higher mean doses of psychotropic medication [10]. They also have less access to psychological therapies and experience worse outcomes from hospitalisation as evidenced by higher rates of relapse and readmission [11]. African-Caribbean groups in the UK are therefore regarded as a ‘high-risk’ population, which is associated with remaining hospitalised two-and-a-half times longer than their White British counterparts and disproportionately being discharged to costly Community Treatment Orders (CTOs) [12, 13].

Against this background, it is perhaps not surprising that African-Caribbeans’ engagement with mainstream mental health services is characterised by fear, mistrust and avoidance [7, 14, 15]. Delayed or non-engagement with services results in a vicious ‘circle of fear’ [14]; involving negative care pathways, coercive treatment and poorer outcomes which reinforces negative perceptions and avoidance of mental health services by African-Caribbean service users and their families [6, 14]. The National Institute for Health and Care Excellence [5] advocates development of specific psychosocial interventions to meet the needs of African-Caribbean people diagnosed with schizophrenia. Recent research also highlights the significance of ethnicity and the crucial role relatives play in pathways to mental health care, in particular for patients with first episode psychosis [16]. Family intervention is one approach which may improve African-Caribbean service users’ engagement with mainstream services, encourage more timely access to care (via less coercive pathways), and improve risk management; thereby enabling better care experiences and outcomes.

### Family intervention and African-Caribbeans

Family intervention (FI) is a psychosocial treatment with a strong evidence base of clinical effectiveness in the management of schizophrenia and other psychoses [17, 18]. The aim of FI is to support service users and their relatives by improving the understanding of severe mental illness and its management and, in so doing, strengthen coping mechanisms and resilience within families. There are a number of approaches to FI for people diagnosed with schizophrenia, common principles include emphasising on FI as part of a total package of care, establishing working alliance with families, addressing family tension, setting reasonable and achievable goals, and focusing on maintaining gains. Core components of FI include psycho-education, problem solving, cognitive appraisal, crisis management and encouraging carers to practice good self-care [17, 18]. Successful engagement with FI is associated with a reduction in relapse and hospital admissions, improvements in medication compliance, social functioning and quality of life, in addition to caregiver outcomes [17, 18].

Previous work by the principal investigator (DE) [19, 20] has influenced our decision to culturally-adapted a widely used cognitive-behavioural model of FI developed by co-applicants Barrowclough and Tarrier [21], which is the model of choice in the NHS Trust where the study will be conducted. Although NICE [5, 22] recommends FI for schizophrenia, implementation and uptake are low [23, 24]. Lack of awareness or understanding of FI might reduce the likelihood of accessing this evidence-based treatment [24, 25], particularly among African-Caribbean groups. However, there is a lack of research on the feasibility of delivering FI to this or other minority ethnic groups [26]. It remains unclear therefore whether the reported benefits of FI are generalisable to African-Caribbean service users and their families [17], particularly given the high rates of associated family disruption and estrangement [27–29]. The aim of this study is to test the feasibility of delivering a novel, culturally appropriate psychosocial intervention within a ‘high-risk’ population to improve engagement and access to evidence-based care.

### Objectives

1. Test the feasibility of delivering Culturally-adapted Family Intervention (CaFI) among African-Caribbean service users in hospital and community settings
2. Test the feasibility of recruiting service users, biological families and ‘family support members’
3. Test the feasibility of delivering the intervention via ‘family support members’ where biological families are not available
4. Assess the acceptability of the intervention to key stakeholders—including service users, their families and mental health professionals
5. Identify outcome measures for future randomised controlled studies and assess the feasibility of collecting them

## Methods/Design

### Design

This study is a feasibility cohort design, incorporating a qualitative component. The research is funded by the National Institute for Health Research (NIHR), Health Service and Delivery Research Programme (HS&DR) (12/5001/62). It was approved by North West—Greater Manchester East National Research Ethics Service (NRES) Ethics Committee (13/NW/0571).

### Sample size

Over 9 months, we aim to recruit and consent a convenience sample of 30 participants via Manchester Mental Health & Social Care Trust (MMHSCT) and community referral. An audit of MMHSCT data via the Trust Clinical Information System (Amigos) at one time point (18.02.2015) indicated that there were 290 service users meeting our inclusion criteria. Excluding those who are too unwell to participate, we estimate Trust staff will approach around 200 potential participants, of whom 150 will be eligible. We conservatively estimate that 1 in 3 will consent to participate in the research. This renders it feasible to recruit *n* = 30 service users, which is sufficient to examine the feasibility of delivering the intervention across a range of service user and family types.

### Participants and recruitment procedures

*Service users* will be recruited via their care teams or self-referral from three settings within MMHSCT, which provides mental health and community services for the inner city areas of Manchester, where the majority of the city’s African-Caribbean population live [30]. Recruiting participants from acute wards, rehabilitation units and community settings facilitates inclusion of service users at differing levels of acuity and chronicity and examination of the feasibility of delivering CaFI across different clinical environments. Our rationale is that, given findings that African-Caribbean people reside in inpatient services for significantly longer periods of time than their White British counterparts [31, 32], working with families in acute as well as community settings might improve engagement and outcome is therefore likely to beneficial.

**Fig. 1:**
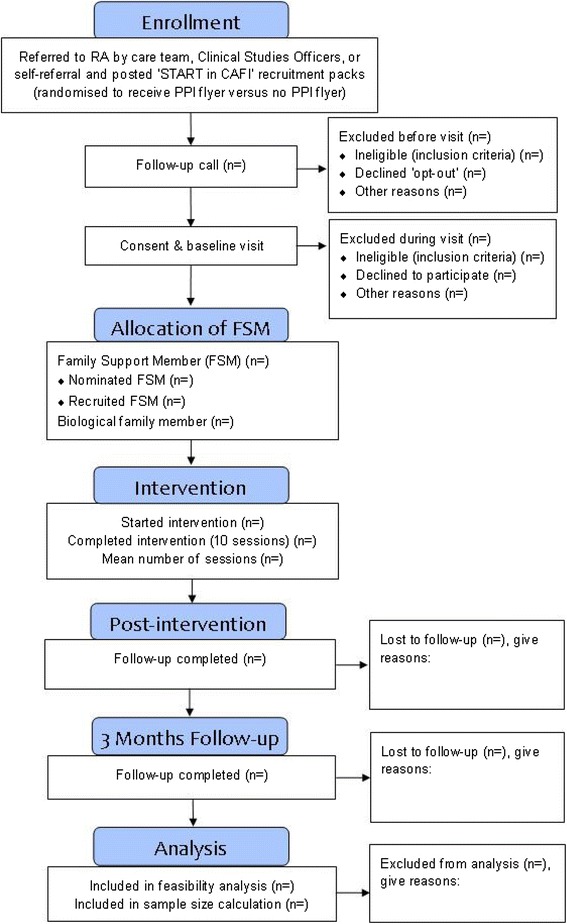
CONSORT diagram illustrating CAFI feasibility study design

Advertisement posters and flyers will be placed in MMHSCT sites that are accessible to service users and in community locations. NIHR Clinical Research Network (CRN) Clinical Studies Officers (CSOs), who have established good relationships with care teams at MMHSCT, will support recruitment by helping to identify and recruit suitable participants. CSOs and research assistants (RAs) will visit teams at CMHTs and inpatient services to inform clinical staff about the study and inclusion criteria. Service users who are well enough (following risk assessment from care coordinator/clinical team) and have capacity to consent and give permission for their contact details to be handed to the research team will receive recruitment packs via the post. The CSO/RA will follow this up with a telephone call (at least 24 h later), where service users will be invited to meet with the RA to receive further information about the study and ask any questions. During the meeting, service users will be asked to self-ascribe their ethnicity (as this is often recorded incorrectly in case notes) and provide written informed consent. Consenting participants will be invited to complete baseline assessments in the initial meeting or an additional meeting. See Fig. 1 for CONSORT diagram detailing the recruitment procedure.

#### START in CaFI

CaFI is participating in the Systematic Techniques for Assisting Recruitment to Trials (START) research programme, which aims to develop and test interventions to improve trial recruitment by supporting the adoption of embedded trials of recruitment interventions across ongoing host studies. Details of the START programme have been published elsewhere [31, 32]. START has received full ethical approval (11/YH/0271). ‘START in CaFI’ is an embedded trial funded by the NIHR, which aims to evaluate whether direct communication of Patient and Public involvement (PPI), via an information leaflet increases service user recruitment into CaFI.

PPI is research being carried out ‘with’ or ‘by’ patients and/or members of the public rather than ‘to’, ‘about’ or ‘for’ them [33]. PPI is deemed good practice as it focuses on the needs and interests of participants [34, 35]. Within CaFI, PPI has been used extensively, with service user and carer consultants forming part of the grant-holding team, in addition to membership of an active Research Advisory Group (RAG) of service users, carers and community members. There is emerging evidence that effective PPI can lead to better participant recruitment [36, 37], and it may increase confidence and trust in research, if potential participants are reassured that other patients have advised its design [38]. However, there is a need to develop a stronger evidence base around the impact of PPI [39]. Better advertising of PPI in research might encourage patient participation.

START in CaFI will test whether directly advertising the PPI in CaFI will improve recruitment into the study. The initial principles underlying the intervention were informed by a review of the literature (Hughes-Morley et. al. 2015) and a workshop (consisting of 27 participants including service users, carers, researchers and members of research ethics committees). The intervention, in the form of a short information leaflet advertising the nature and function of the PPI in CaFI, has been developed by the CaFI RAG to meet the needs of African-Caribbean people diagnosed with schizophrenia and their families. Professional graphic design and user-testing has optimised readability and impact. The leaflet will complement the PIS.

Service users will be randomised to receive the leaflet alongside the standard PIS, or the standard PIS alone (provided in the posted recruitment packs), to test whether receiving the leaflet is associated with higher levels of recruitment into CaFI.

*Biological family members* will be recruited via service users (and their care teams) or through self-referral. Posters and flyers advertising the study will be placed in appropriate locations in MMHSCT settings such as visiting/relatives’ rooms and noticeboards and community settings such as community centres, carer support groups and churches. Previous PPI work with African-Caribbean communities indicates that churches play an important role in terms of community cohesion and knowledge transfer. Working with ‘Black-majority’ churches is therefore an innovative and culturally-sensitive approach to recruitment within this ethnic group.

Where service users are recruited first, we will seek their permission to contact their families and invite them to participate. Interested family members may also self-refer to the research team directly or via care teams. Permission will be sought for the CSO/RA to contact the service user to see if they are willing to be approached and learn more about the study (following the above process for service user recruitment). Family members will be posted the PIS detailing the nature of the research and their potential involvement. The CSO/RA will contact the family member by telephone (at least 24 h later) to arrange an initial appointment, where they will be receive information about the study and have the opportunity ask questions. Informed consent and baseline assessments will be conducted for those opting in, as per procedure outline above.

*Family support members* (FSM) will work alongside service users who would like to receive the therapy but do not have contact with their biological families, acting as ‘proxy family’ members. This novel aspect of the study was devised to facilitate access to our intervention. We know that serious mental illness can negatively impact the social networks of all people [40], irrespective of ethnicity. However, as African-Caribbeans have much longer duration of untreated illness than other groups and more fragmented networks, they are likely to experience high levels of family burden and disrupted family relationships. Research from our New Zealand collaborators found that ‘trusted individuals’ from the community could work effectively with service users thus enabling them to access family intervention [41].

They will be recruited in one of two ways:

1. *Service-user nominated*: Service users will be asked to nominate trusted individuals, such as friends, support workers or a local pastor. The CSO/RA will confirm with service users that individuals they wish to nominate as FSMs have been made aware of the study and are happy to be approached. Recruitment, consent and baseline assessments will be conducted following the procedure for biological family members as above.
2. *Recruited*: Service users who are unable to nominate anyone but wish to participate will be given the opportunity to select a FSM from a pool of community volunteers who have been specifically recruited for this study. Nine FSMs have been successfully recruited using extensive public engagement via local media (community radio, newspaper), posters and flyers and by delivering presentations at third sector organisations and community settings frequented by large numbers of African-Caribbeans, such as Black-majority churches. Potential FSMs met with the RA to determine their suitability and were fully briefed about the study (PIS) before providing informed consent to undertake the role.
  Recruited FSMs have been given honorary contracts to work at Trust sites, which involved obtaining Disclosure and Barring Service checks, signing confidentiality agreements and completing occupational health checks. They received one-day Cultural Competency training delivered by *Just Psychology* [44] (Feb 2015) and a 2-h training session facilitated by the PI and RA on the research protocol and the FSM role (Mar 2015). Each FSM wrote a brief biography and described their interest in undertaking the role. This information will be given to service users to help them decide which FSM they would like to work with. Once the FSM has been matched to a service user, an initial meeting will be scheduled to conduct baseline assessments.

### Inclusion/exclusion criteria

*Service users* must be of African-Caribbean descent (including those who self-identify as ‘Black-British’, ‘African-Caribbean’ or ‘mixed’ African-Caribbean but who have at least one African-Caribbean parent or grandparent). They must have a diagnosis of schizophrenia or related diagnoses (ICD F20-29) [43] and be receiving treatment through psychiatric acute or rehabilitation inpatient services or community services within MMHSCT. Service users must be 18 or older, assessed by key workers as having the capacity to consent and participate and have sufficient understanding of English language to complete measures. Those having significant cognitive impairment implicated in aetiology (e.g. organic disorder) or assessed as presenting high risk to self or others by care teams will be excluded.

*Biological family members* and *FSMs* need not be of African-Caribbean origin but must be at least 18 years old and have sufficient understanding of English language to be able to give written, informed consent and complete measures. Recruited FSMs will need to successfully complete relevant checks as detailed above.

### The intervention: Culturally-adapted Family Intervention (CaFI)

#### Development of CaFI

CaFI is a culturally adapted version of the structured, cognitive-behavioural model of FI developed by Barrowclough and Tarrier (1992) comprising a psycho-educational component and cognitive-behavioural skills for stress-management, coping and problem solving. FI was largely influenced by pioneering research showing that people with schizophrenia are more likely to experience relapse when they experience high levels of hostility, criticism or emotional over-involvement within the family [44]. Working in collaboration with the family to help them to understand schizophrenia and related psychoses and tackle their problems has been shown to alleviate stress and reduce the risk of relapse [45–47].

Cultural adaptation of Barrowclough and Tarrier’s FI (1992) for the production of a CaFI therapy manual (Edge, Degnan, Berry, Drake, Barrowclough and Abel, in preparation) has been informed by three main sources. First, we undertook a literature review of theoretical and intervention papers to identify important factors for successful cultural-adaptation of psychosocial interventions in schizophrenia. Second, we conducted qualitative research to determine key stakeholders’ perspectives on how to culturally adapt Barrowclough and Tarrier’s (1992) model of FI to meet the particular needs of African-Caribbean families. Stakeholders participated in four focus groups: three separate focus groups comprising service users (*n* = 10), carers and advocates (*n* = 14), and health professionals (*n* = 7) and a fourth ‘mixed’ focus group of purposefully selected individuals from the three original groups (*n* = 11). The purpose of the ‘mixed’ group was to resolve any disagreements and validate findings from the initial focus groups. Third, an expert Consensus Conference comprising 22 ‘experts’ drawn from a national sample, including experts by experience (carers, family members, service users) and experts by profession (academics, health professionals, police and service managers), was conducted to synthesise data from the literature and focus groups, and to identify and agree on essential elements for culturally-adapting FI (CaFI) for African-Caribbeans. The process of culturally-adapting the intervention is detailed in a forthcoming publication. Key aspects of CaFI are outlined below.

#### Ethos and mode of delivery

Focus group participants were unanimous that, whilst culturally adapting the content of the intervention was important, the most fundamental adaptation needed to be reflected in the way CaFI is delivered. Participants espoused a ‘recovery-focused’ and highly collaborative or ‘shared learning’ approach where therapists acknowledge families’ strengths and are willing to learn from them; especially in relation to culture and models of mental health/illness. Participants emphasised that, from their standpoint, recovery should be regarded as self-acceptance and achieving a good quality of life versus elimination of symptoms. In line with good clinical practice, individualised formulations must be used to understand problems and develop solutions tailored to meet the needs of individual families.

#### Therapists and training

Each CaFI session will be delivered by a lead therapist (NHS Agenda for Change Band 7) and a co-therapist (NHS Band 4). Three pairs of therapists (*n* = 6) have been recruited to deliver 300 h of CAFI to 30 families (30 × 10 hourly sessions); equating to 100 h of therapy per therapist over the 12-month intervention period. Therapists have come from a range of professions, including clinical psychology, social work, mental health nursing and occupational therapy. Lead therapists have relevant training and skills in CBT and FI, either directly through their current profession or through advanced training in psychosocial interventions for psychosis. Co-therapists are current mental health support workers or assistant psychologists. Therapists deliver CaFI as part of their current caseload—reflecting our ultimate goal to embed FI within NHS services. To ensure familiarity with our ‘ethos of delivery’, all six therapists have received 2 days of training in cultural competence (delivered by *Just Psychology* [42]) and family working skills (delivered by *Meriden Family Programme* [48]). Key components of therapists’ training included the following: (i) core competences to work effectively with families experiencing schizophrenia and psychosis [49]; (ii) current legislation and how it relates to clinical practice (such as The Equality Act, 2010) [50], and NHS Knowledge Skills Framework requirements [51]; (iii) cultural awareness and family work practice (for example, the potential impact of culture and family experience on delivery of evidence-based FI components and cultural diversity within African-Caribbean communities); (iv) relationship between racism, discrimination, adversity and mental health; and (v) the significance of power and prejudice in building trusting therapeutic relationships. Therapists also received half-day training in the CaFI manual facilitated by the research team [PI, RA and clinical supervisor] to engender confidence in delivering the sessions. Therapists will receive bi-weekly hourly supervision from an experienced clinical psychologist on the research team. To share good practice and highlight common issues within sessions, there will also be group supervision sessions. The frequency of these will be agreed with the therapists and depend on need.

#### Manual content

The CaFI intervention includes five key components:

1. *Engagement and assessment [two sessions]*
  In the development phase, participants stressed the importance of taking time to build trust given the history of negative relationships between African-Caribbeans and statutory mental health services. Accordingly, in these initial sessions, the emphasis will be on trust-building and engagement, developing a positive therapeutic relationship, and establishing working therapeutic alliance between family members. Therapists will undertake a thorough assessment of the family; identifying strengths and resources as well as a formulation of needs. Additionally, explanation of the intervention is provided (including proposed structure and purpose and potential benefits to the family), problems identified and prioritised based on the family’s wishes and formulation, realistic SMART (specific, measurable, attainable, relevant, timely) goals and expectations for future session set.
2. *Shared learning [two sessions]*
  ‘*Shared learning*’ is a collaborative approach to psycho-education designed to facilitate engagement and alliance. This approach allows the therapist, relatives and service user to learn from each other’s experiences and acquire knowledge that will lead to more beneficial ways of managing difficulties related to schizophrenia and psychosis in the family. This is an important aspect of cultural-adaptation for this ethnic group; particularly in terms of addressing illness beliefs and explanatory models. In conjunction with initial assessment, these sessions lay the groundwork for other components of the intervention and behaviour change.
3. *Communication [two sessions]*
  The ‘*communication*’ sessions support service users and relatives to communicate more effectively with each other and with service providers. The emphasis on understanding ‘*how the system works*’ and developing communication and advocacy skills was regarded by participants as a highlighting culturally-specific difference between the needs of African-Caribbeans and other groups. Additionally, as when working with other groups, therapists will be expected to model and positively reinforce effective communication skills, including establishing ground rules for good communication from the outset. The specific communication skills to be addressed with each family will be decided collaboratively with the family members based on the initial assessment and formulation. Developing good communication skills lays down an important foundation for subsequent sessions on problem solving and goal setting.
4. *Stress management, coping and problem Solving [two sessions]*
  These sessions focus on helping both service users and relatives to manage current stressors through joint problem-solving or other ways of coping that may help reduce family tension. These sessions flow from and complement the previous session on communication. As communication difficulties can be a significant source of stress for families, improved communication can help families work more collaboratively to solve problems.
5. *Maintaining gains and staying well [two sessions]*
  The aim of the final two sessions is to review and consolidate the material that has been covered over the preceding sessions and to develop a plan for staying well as a family and reducing the risk of further relapse. To make these sessions more culturally-appropriate, the focus will be on recovery, emphasising strengths and aspirations, and the importance of having an agreed crisis plan to improve care pathways by reducing the likelihood of police involvement and coercive care. Therapists will establish what recovery means for each family; help them set realistic expectations for positive, on-going change; and address any difficulties the family might experience with endings. Providing the family with a ‘goodbye letter’ is an important opportunity for the therapist to give positive feedback on the family’s strengths and hard work in therapy; thereby reinforcing therapeutic engagement and alliance.

#### Duration and intensity of therapy

CaFI is designed to be conducted over ten sessions as recommended by NICE (2014). To allow families to work on issues outside the session and facilitate potential absence or illness; CaFI will be delivered over approximately 20 weeks. The pace of the sessions will depend upon the needs of the family and may be arranged weekly initially to facilitate engagement and cover core components but subsequently, towards the end of therapy, might be reduced to fortnightly or longer. Each session will last around 1 h (in practice, 1.5 h with an additional 30-min preparation and debriefing).

## Data collection

### Quantitative data

#### Feasibility and acceptability of recruitment and delivery

We will assess the feasibility of delivering CaFI, including attendance, attrition (number of drop-outs at each time point), and retention (the proportion of participants who complete therapy sessions). Therapists will record session attendance and retention data to measure feasibility of delivery (i.e. location, duration, intensity, attendees). Participants (service users and biological family members/FSMs) will be asked to complete evaluation/feedback forms at the end of each session to monitor the acceptability of the intervention.

We shall assess the feasibility of undertaking research on CaFI, by studying recruitment (the proportion of eligible participants consenting to join the study) and completeness of outcome measurement. Data will be collected on reasons for ineligibility and non-consent; including anonymous information on gender, ethnicity and date of birth for those who are approached but do not consent to take part. We shall record and compare recruitment rates across different referral sites (inpatient, community, third sector) and sources (CRN, research team, self-referral, clinical referral).

### Outcome measures for future RCT design

To identify outcome measures for future randomised controlled trials (RCTs) and assess the feasibility of collecting them; participants (service users and biological family members/FSMs) will complete a range of quantitative outcome measures at baseline, post-intervention and 3-month follow-up. These will be conducted by trained RAs who are independent from the delivery of the therapy. Where participants leave the intervention early, we shall attempt to gather outcomes at exit and 3-month follow-up. Assessing feasibility of collecting these measures will be important to inform a later trial and economic evaluation.

#### Socio-demographic questionnaire

A self-report socio-demographic questionnaire to collect data on key variables such as age, gender, ethnic group and religion will be completed by service users, family members and FSMs. Additional questions for service users will include diagnosis, relationship with the family member/FSM, length of time since first contact with services, inpatient history and medication.

### Service user and family outcome measures

#### Psychosis symptom severity (service users)

Tables 1 and 2 show the patient and family measures used in the study and outlines the data collection schedule. The *Positive and Negative Syndrome Scale* (PANSS) [52] is a widely used 30-item semi-structured interview designed to assess positive, negative and general symptoms in service users with schizophrenic spectrum diagnoses. The PANSS has good psychometric properties of reliability and validity and is sensitive to change [52, 53]. Two trained RAs will rate the PANSS and we will report inter-rater reliability.

**Table 1:** Patient assessment schedule

Assessment tool	Brief description	Time point			
		Duration (min)	Baseline	Post-CaFI	3-month follow-up
Socio-demographic	Socio-demographic	5	x		
PANSS	Symptoms	30–40	x	x	x
PSP	Personal and social functioning	5	x		x
PCS	Perceived criticism	5	x	x	x
Brief-IPQ	Illness beliefs	5	x		
EQ-5D	Economic evaluation	5	x	x	x
WAI—short form	Working alliance/engagement	5	x	x	x
Qualitative interview	Acceptability and feasibility	30–45			x
Total time burden			60–80	45	80–95
Session feedback forms	Acceptability	5 min end of each session			
WAI—short form Session 3	Therapeutic alliance	5 min complete during session 3			
Relapse	Case notes 40 weeks before, during and 40-week post-intervention	0 min—undertaken by independent review at 3-month FU

**Table 2:** Family member/family support member (FSM) assessment schedule

Assessment tool	Brief description	Time point			
		Duration (min)	Baseline	Post-CaFI	3-month follow-up
Socio-demographic	Socio-demographic	5	x		
GHQ—short form	Stress/burden	5	x	x	x
KAPI—relatives^a^	Knowledge about psychosis	15–30	x	x	x
Brief-IPQ^a^	Illness beliefs	5	x		
EQ-5D	Economic evaluation	5	x	x	x
Qualitative interview	Acceptability and feasibility	30–45			x
Total time burden			35–50	25–40	55–70
WAI—short form Session 3	Therapeutic alliance	5 min complete during session 3			
Session feedback forms	Acceptability	5 min end of each session			

^a^Biological family members and nominated FSMs only (not recruited FSMs)

#### Social functioning (service users)

The *Personal and Social Performance Scale* (PSP) [54] is a 100-point, observer-rated, single-item scale. The scale measures social functioning across the past month in four areas: socially useful activities (including work and study, personal and social relationships, self-care and disturbing and aggressive behaviours. It is reliable, valid and sensitive to change and correlates with PANSS scores [55]. Ratings will be made by the RA on the basis of service users’ reports of symptoms, service users’ behaviour during PANSS interviews, and reports from care staff and significant others. PSP data will be collected at baseline and 3-month follow-up only.

#### Perceived criticism (service users)

The *Perceived Criticism Scale* (PCS) [56] is a 4-item self-report measure of service user perceptions of relatives’ criticism. It provides an efficient way of assessing negative aspects of the psychosocial environment. The PCS is a reliable and valid measure [57, 58] and perceived criticism has been shown to predict symptom course, treatment outcome and relapse in schizophrenia [44, 57]. Service users will be asked to complete this measure in relation to their family member/FSM. If there is more than one biological family member/FSM taking part in the intervention, the service user will be asked to rate the person who is currently the most important to them and with whom they share the closest relationship.

#### Illness beliefs (service users, biological family members/nominated FSMs)

A 12-item modified version of the Brief Illness Perception Questionnaire (Brief-IPQ) [59] will be used to assess illness perceptions in service users and family members/nominated FSMs at baseline. The Brief-IPQ, like the original IPQ (Addington, 2003) from which it was derived, were designed for physical health problems but can be adapted for mental health problems (Lobban et al. 2005; Lobban et al. 2013). Modifications for this study are coherent with previous adaptations for mental health (Lobban et al., 2013) and include replacing the word ‘illness’ with ‘mental health problems’ and adding three items that assess the following: personal effort (how much effort the individual is making to help them get well); cause internal (the extent to which the symptoms are caused by the individual’s behaviour); and self-blame (the extent to which the individual is to blame for the mental health problems). The Brief-IPQ has demonstrated good reliability and validity [59] and has previously been used in psychosis research e.g. [60–62].

#### Knowledge about psychosis (biological family members/nominated FSMs)

The *Knowledge about Psychosis Interview* (KAPI) [63] is a revised version of the *Knowledge about Schizophrenia Interview* (KASI) [21]. The KAPI will not be conducted with recruited FSMs at baseline as they will have limited knowledge of the service user’s problems before the intervention. As KASI and KAPI are culturally-insensitive and use outdated language; we are currently developing and validating two updated versions of these instruments: (1) the Knowledge About Psychosis (KAP) questionnaire, for use in a general population sample; and (2) the Culturally-adapted Knowledge About Psychosis (CaKAP) questionnaire, which has been adapted for the African-Caribbean community (Degnan et al., in prep). It is anticipated that the questionnaire(s) will be available for use in future RCTs.

#### Family stress/burden (biological family members/FSMs)

The 12-item *General Health Questionnaire* (GHQ-12) [64] is one of the most widely used and valid measures of emotional distress and is frequently used to detect the risk of psychiatric morbidity. It will be used as a measure of burden and general stress among family members and FSMs.

#### Economic evaluation (service users, biological family members/FSMs)

The *EQ-5D-5L* [65] is a generic preference-based self-report measure of health-related quality of life (HRQoL), which covers five domains: mobility, self-care, usual activities, pain/discomfort and anxiety/depression. Individuals’ responses to the EQ-5D-5L can be used to calculate a single index utility value from a tariff derived from UK population-based valuation studies. These utility values are used as the quality adjustment component in the calculation of quality-adjusted life years (QALYs) within economic evaluations. The EQ-5D-5L has been validated in diverse populations [66] and is recommended by NICE [67].

#### Working alliance (service users, family members/FSMs)

The *Working Alliance Inventory* (*WAI*)*-short-form* [68] is a 12-item self-report measure of the quality of staff-service user relationships and comprises three subscales; agreement on goals, agreement on tasks and emotional bond. The WAI short-form has good psychometric properties [69]. Working alliance has also been shown to influence outcome in therapy [70–72]. Service users will complete the WAI in relation to their key worker at the three assessment time points. Service users and family members/FSMs will also complete the WAI in relation to the therapist dyad at the end of session 3.

#### Relapse rates

Reduction in relapse will be analysed using two recognised methods [73]: (1) number and duration of inpatient admissions identified from hospital notes and (2) number and duration of exacerbations of symptoms lasting longer than 2 weeks and requiring a change in service user management such as increased observation and/or medication change by clinical team as assessed by hospital case notes. Where symptom exacerbation precedes hospitalisation, only one relapse will be recorded. Discharge rates and hospital admissions will be recorded and whether service users were discharged to higher or lower intensity services. For each participant, case notes will be examined at three time points: (i) the period 40 weeks prior to the intervention, (ii) the duration of the intervention (20 weeks) and (iii) 40-week post-intervention. Relapse will be rated retrospectively by two independent reviewers at the end of the study (at 3-month follow-up).

### Staff outcome measures

#### Working alliance (key workers, therapists)

Staff outcomes and schedule of data collection are presented in Tables 3 and 4. The staff version of the *WAI-12* [68] will be completed by key workers in relation to the service user at the three assessment time points. Items are identical to those of the service user measure but are reworded to reflect the perspective of the staff members. The lead and co-therapists will also complete the WAI in relation to the service user and each family member/FSM at the end of session 3. The WAI has previously been used with key workers and therapists [74–76].

**Table 3:** Key Worker Assessment Schedule

Assessment tool		Time point			
		Duration (min)	Baseline	Post-CaFI	3-month follow-up
Referral info. and demographic	Socio-demographic	10	x		
WAI—short form	Working alliance	5	x	x	x
SES	Service engagement	5	x	x	x
Qualitative interview (*n* = 10)	Acceptability and feasibility	30–45			x
Total time burden			20	10	40–55

**Table 4:** Therapist assessment schedule

Assessment tool		Time point			
		Duration (min)	Baseline	Post-CaFI	3-month follow-up
Qualitative interview	Acceptability and feasibility	30–45			x
WAI—short form session 3	Therapeutic alliance	5 min complete during session 3			
Total time burden		50 min			

#### Service engagement (key workers)

The *Service Engagement Scale* (SES) [77] is a 14-item self-report measure assessing participants’ engagement with services from a key worker perspective. The measure has four subscales; availability; collaboration; help-seeking; and treatment adherence. The SES has been validated in a psychosis sample, with evidence of good psychometric properties [77].

### Fidelity study

The CaFI fidelity measure comprises a modified version of the subscale of the Cognitive Therapy Scale for Psychosis (CTS-PSY) (Haddock et al. 2001), which has been adapted for this study to account for the presence of two therapists and the relatives. Six items will be used: agenda setting, feedback, understanding, interpersonal effectiveness, collaboration, homework and quality of CBT techniques. The second subscale includes components that map directly onto the CaFI therapy manual along with two additional items from the Family Interventions in Psychosis-Adherence Scale (FIPAS) (Onwumere et al. 2009): reducing criticism and conflict and reducing over-involvement. Fidelity will be assessed by independent ratings of 10 % of randomly selected sessions. Treatment fidelity and quality will also be monitored via discussion of audio-recordings of sessions in supervision.

### Qualitative data

Qualitative interviews of all consenting participants will be conducted post-intervention (within the 3-month follow-up period) to assess the acceptability of the intervention to service users, biological families/FSMs and therapists. In addition, we shall interview a sample of key workers (*n* = 10) purposively selected to achieve-variation in gender, profession, length of experience, clinical setting, participants’ retention in CaFI (i.e. those who completed the sessions and those who withdrew) and familial relationship type (biological family member/FSM). Data will be collected using topic guides specifically designed to explore participants’ perspectives on different aspects of the study such as the following: (i) taking part in research; (ii) content and delivery of sessions; (iii) usefulness, cultural-appropriateness and accessibility of intervention and materials; (iv) barriers/facilitators to implementation; (vi) training/supervision; (vii) personal benefits; (viii) delivery via FSMs and therapists. Inclusion of open-ended questions in a semi-structured interview format will enable us to explore aspects of the intervention that participants particularly liked/disliked and areas which they think should have been improved.

Participants (service users and biological family members/FSMs) who withdraw from the intervention early will be asked to complete a withdrawal form and the above qualitative interview, with redundant items removed. Additional questions will be asked about why they left early and what could have been done differently to facilitate retention.

### Data analysis

*Quantitative analyses* of demographic information and quantitative elements of the questionnaires will be presented using descriptive statistics [78]. We shall also present descriptive statistics on recruitment, consent, adherence and attrition, including exploratory analysis of factors affecting adherence and attrition'. We shall examine the characteristics of the various outcome measures to consider which might be most appropriate in a future trial and estimate variability to inform sample size calculation. We are aware of the problem of loss to follow-up in mental health trials. We shall prepare for this in a future trial by examining outcomes at points of departure from the trial. Quantitative data will be analysed using STATA 14 [79].

*Qualitative data* will be digitally-recorded, transcribed, checked for accuracy and analysed using thematic analysis [80]. NVivo-10 [81] will support data management and analysis. Thematic analysis is a useful and flexible method for identifying and describing themes (or patterns of meaning) from rich qualitative data [80]. The themes will be derived inductively to an extent but coding and analysis across interviews will be guided by study objectives. Coding will therefore be an iterative process, developed over time by moving back and forth through the following phases of analysis to ensure themes are developed in a methodologically rigorous way [80]: data familiarisation, searching for themes; reviewing themes; and defining and naming themes.

## Discussion

Despite decades of research and major Department of Health investment aimed at ‘Delivering Race Equality’ in mental health [15], NICE concluded that the care and treatment of African-Caribbeans diagnosed with schizophrenia remains in crisis [5]. Service responses are inconsistent and often ineffective. NICE guidance [5] highlights an urgent need to develop culturally appropriate, evidence-based interventions for African-Caribbeans because, compared with other studied groups, evidence suggests they experience significantly higher levels of morbidity, inferior access to care and worse outcomes [10, 14]. Proactive, recovery-based, family-centred approaches improve risk management and outcomes by identifying early warning signs and addressing them [82].

In response to this, we have developed a potentially ground-breaking innovation to meet the specific needs of African-Caribbean people thus improving access and reducing inequity in clinical outcomes for members of this ethnic group. Not only have we culturally-adapted an existing well-established clinical FI [83], but we have also engaged directly with local African-Caribbean stakeholder communities and successfully recruited ‘proxy family’ members to enable service users, who might otherwise be excluded due to lack of family contacts, to receive FI. In so doing, we have overcome one of the greatest challenges to developing and testing our intervention, namely engaging with a so called ‘hard-to-reach’ community with a long history of mistrust of statutory mental health services and high levels of community stigma. Engaging community members in delivering the intervention potentially addresses an important access barrier for African-Caribbeans who are especially likely to experience family disruption [29]. Other important and novel aspects of the study include testing the intervention in both hospital (acute and rehabilitation wards) and community settings and making it available to individuals on CTOs. We shall test the feasibility of implementing the evaluation and evaluating its acceptability among African-Caribbean service users, their families and service stakeholders.

Successful implementation of CaFI will facilitate and improve engagement in services for African-Caribbean people with schizophrenia diagnoses (ICD F20-29), thus improving access to a range of evidence-based interventions designed to improve patient- and service-led outcomes. Successful implementation of CaFI in the African-Caribbean community, with its innovative solution to therapeutic engagement of disrupted family units and people living with severe mental illness, offers a means of improving access to evidence-based care and clinical and life outcomes for other socially excluded and marginalised communities. This is especially important in the current climate of refugee and asylum-seeker migration, which means that the landscape of delivering mental health care for minority populations is changing rapidly. It is therefore increasingly imperative that new solutions are found to tackle persistent, ethnically-based inequities in mental health care, which are likely to represent ongoing challenges for policy makers, commissioners and service providers.

## Abbreviations

BME, Black and Minority Ethnic; CaFI, Culturally-adapted Family Intervention; CaKAP, Culturally-adapted Knowledge about Psychosis; CRN, Clinical Research Network; CSOs, Clinical Studies Officers; CTOs, Community Treatment Orders; CTS-PSY, Cognitive Therapy Scale for Psychosis; FI, Family Intervention; FIPAS, Family Interventions in Psychosis-Adherence Scale; FSM, Family Support Members; GHQ-12, General Health Questionnaire; GPs, General Practitioners; HS&DR, Health Service and Delivery Research; IPQ, Illness Perception Questionnaire; KAP, Knowledge about Psychosis; KAPI, Knowledge about Psychosis Interview; KASI, Knowledge about Schizophrenia Interview; MMHSCT, Manchester Mental Health and Social Care Trust; NICE, National Institute of Health and Care Excellence; NIHR, National Institute for Health Research; NRES, National Research Ethics Service; PANSS, Positive and Negative Syndrome Scale; PCS, Perceived Criticism Scale; PIS, Participant Information Sheet; PPI, Patient and Public Involvement; PSP, Personal and Social Performance Scale; QALYs, quality-adjusted life years; RAG, Research Advisory Group; RAs, research assistants; RCT, Randomised Controlled Trials; SES, Service Engagement Scale; SMART, Specific, Measurable, Attainable, Relevant, Timely; SMI, severe mental illness; START, Systematic Techniques for Assisting Recruitment to Trials; WAI, Working Alliance Inventory
